# The role of professionals' associations under extraordinary situations: Contingency, capacity, and collaboration

**DOI:** 10.1192/j.eurpsy.2021.45

**Published:** 2021-08-13

**Authors:** K. Başar

**Affiliations:** 1 Secretary General, Psychiatric Association of Turkey, Ankara, Turkey; 2 Department of Psychiatry, Hacettepe University Faculty of Medicine, Ankara, Turkey

## Abstract

COVID-19 pandemic, with its profound effects on almost every sphere of individual and social life, is a significant challenge and threat to mental well-being. Although mass disasters with similar traumatic effects are not exceptional incidents globally, the rate of the spread of infection, the scale of the effects of the disease and precautions, and uncertainty concerning the nature, prevention from, and treatment of the disease render the psychosocial effects unique. As it is the case for the individual’s response to the stressful events, the psychiatric communities initially addressed this challenge by adapting their usual responses to mass trauma, through their capacity acquired from earlier experiences and training. Although the response to the pandemic is expected to be orchestrated by the public authorities, in many countries, either the administration was not sufficiently cognizant of the psychosocial consequences of the pandemic, or the health-care system was unable to function properly due to the excessive burden. Therefore, the associations of mental health professionals, with varying degrees of preparedness to cope with such a challenge, had to recruit their full resources. As many associations worldwide did, the Psychiatric Association of Turkey prepared written and audiovisual resources for psychiatrists, health professionals, and the general population related to the mental health effects of the pandemic and precautions, often including strategies to cope with stress-related difficulties. Many associations, also provided distant-access psychological support to health-care workers on the frontlines and the general population. These were achieved through a fast-organized collaboration among its members and between associations worldwide.

**Disclosure:**

No significant relationships.

